# LC/MS Profiling and Gold Nanoparticle Formulation of Major Metabolites from *Origanum majorana* as Antibacterial and Antioxidant Potentialities

**DOI:** 10.3390/plants11141871

**Published:** 2022-07-18

**Authors:** Ahmed H. El-Ghorab, Fathy A. Behery, Mohamed A. Abdelgawad, Ibrahim Hotan Alsohaimi, Arafa Musa, Ehab M. Mostafa, Hamud A. Altaleb, Ibrahim O. Althobaiti, Mohamed Hamza, Mohammed H. Elkomy, Ahmed A. Hamed, Ahmed M. Sayed, Hossam M. Hassan, Mahmoud A. Aboseada

**Affiliations:** 1 Department of Chemistry, College of Science, Jouf University, Sakaka 72341, Saudi Arabia; aghorab@ju.edu.sa (A.H.E.-G.); ehalshaimi@ju.edu.sa (I.H.A.); 2 Department of Pharmacognosy, Faculty of Pharmacy, Mansoura University, Mansoura 35516, Egypt; fathy.behery@riyadh.edu.sa; 3 Department of Pharmacy, College of Pharmacy, Riyadh Elm University, Riyadh 11681, Saudi Arabia; 4 Department of Pharmaceutical Chemistry, College of Pharmacy, Jouf University, Sakaka 72341, Saudi Arabia; 5 Department of Pharmacognosy, College of Pharmacy, Jouf University, Sakaka 72341, Saudi Arabia; akmusa@ju.edu.sa (A.M.); emmoustafa@ju.edu.sa (E.M.M.); 6 Department of Chemistry, Faculty of Science, Islamic University of Madinah, Madinah 41477, Saudi Arabia; haltaleb@iu.edu.sa; 7 Department of Chemistry, College of Science and Arts, Jouf University, Sakaka 72341, Saudi Arabia; ioalthobaiti@ju.edu.sa; 8 Department of Biology, College of Science, Jouf University, Sakaka 72341, Saudi Arabia; mhabdelhameed@ju.edu.sa; 9 Department of Pharmaceutics, College of Pharmacy, Jouf University, Sakaka 72341, Saudi Arabia; mhalkomy@ju.edu.sa; 10 National Research Centre, Microbial Chemistry Department, 33 El-Buhouth Street, Dokki, Giza 12622, Egypt; ahmedshalbio@gmail.com; 11 Department of Pharmacognosy, Faculty of Pharmacy, Nahda University, Beni-Suef 62513, Egypt; ahmedpharma8530@gmail.com; 12 Department of Pharmacognosy, Faculty of Pharmacy, Beni-Suef University, Beni-Suef 62513, Egypt; hossam.mokhtar@nub.edu.eg

**Keywords:** *Origanum majoranum* L., metabolomics, 7-methoxyepirosmanol, antimicrobial, antioxidant potential, gold nanoparticles, high features

## Abstract

*Origanum majoranum* L. is a *Lamiaceae* medicinal plant with culinary and ethnomedical applications. Its biological and phytochemical profiles have been extensively researched. Accordingly, this study aimed to investigate the chemical composition and the antibacterial and antioxidant properties of *O. majoranum* high features, as well as to search for techniques for activity optimization. A metabolomics study of the crude extract of *O. majoranum* using liquid chromatography-high-resolution electrospray ionization mass spectrometry (LC ± HR ± ESI ± MS) was conducted. Five fractions (petroleum ether, dichloromethane, ethyl acetate, n-butanol, and aqueous) were derived from the total extract of the aerial parts. Different chromatographic methods and NMR analysis were utilized to purify and identify the isolated phenolics (high features). Moreover, the antimicrobial, antibiofilm, and antioxidant activity of phenolics were performed. Results showed that metabolomic profiling of the crude extract of *O. majoranum* aerial parts revealed the presence of a variety of phytochemicals, predominantly phenolics, resulting in the isolation and identification of seven high-feature compounds comprising two phenolic acids, rosmarinic and caffeic acids, one phenolic diterpene, 7-methoxyepirosmanol, in addition to four flavonoids, quercetin, hesperitin, hesperidin, and luteolin. On the other hand, 7-methoxyepirosmanol (OM_1_) displayed the most antimicrobial and antioxidant potential. Such a phenolic principal activity improvement seems to be established after loading on gold nanoparticles.

## 1. Introduction

*Origanum* is one of 200 genera of the *Lamiaceae* family, containing 3500 species worldwide. The vast majority of species are fragrant and grow naturally in the Mediterranean region [1,2,3,4]. The genus is characterized by large morphological and chemical diversity. The morphological differences within the genus result in dividing the genus into 10 divisions, each including 49 taxa (species, sub-species, and varieties) [5,6,7]. *Origanum majoranum* L., also known as *Majorana hortensis* Moench, is a tender perennial herb in the genus “*Origanum*” [8]. It is sometimes referred to as sweet marjoram and is endemic to Cyperus, Antolia (Turkey), and has been naturalized in sections of the Mediterranean region, particularly Egypt [9]. It is grown for its flavor and aroma throughout the world, including in India, France, Hungary, and the United States. Marjoram was initially used as an antiseptic by Hippocrates. It is a popular home remedy for chest infections, sore throat, cough, rheumatoid arthritis, mental disorders, epilepsy, cardiovascular diseases, sleeplessness, skincare, flatulence, and stomach problems. [10,11,12]. Pharmacologically, marjoram was evaluated for its antioxidant, anti-anxiety, anti-convulsant, anti-diabetic, anti-gout, anti-mutagenic, anti-ulcer, antibacterial, antifungal, and antiprotozoal activities [13,14,15,16,17]. Sweet marjoram has a pungent, spicy, and pleasant aroma and flavor. Analysis of the essential oil reported volatile constituents as major metabolites, predominantly terpinen-4-ol, cis-sabinene hydrate, *p*-cymene, sabinene, and trans-sabinene hydrate [4,18]. Various phytochemical tests on ethanolic extracts revealed the presence of terpenoids such as oleanolic acid and ursolic acid [8,19], flavonoids, namely apigenin, arbutin, catechin, rutin, hesperidin, and amentoflavone, phenolic acids, such as rosmarininc acid, caffeic acid, and coumaric acid, and tannins such as gallic acid [20,21,22].

In natural product research, dereplication has been widespread, allowing for rapidly identifying known metabolites in complex combinations. [23,24]. It is significantly easier to screen samples for known natural chemicals with LC-MS dereplication and subsequent database searches, such as Reaxys online database and the Dictionary of Natural Products (DNP) on DVD [25,26]. It reduces the likelihood of re-isolation redundancy in natural product discovery methods and saves time. Metabolomics also thoroughly examines chemicals in a biological system under a specific set of conditions [27]. The metabolome is most intimately related to the phenotype at the molecular level, providing insight into biological activities [28].

This study intends to investigate the chemical and biological profiles of the plant as mentioned above as part of our ongoing research on it. In this approach, the secondary metabolites of *Origanum majoranum* will be initially assessed and dereplicated utilizing metabolomic analysis via liquid chromatography combined with high-resolution electrospray ionization mass spectrometry (LC-HRESIMS). Subsequently, we assess datasets for correlations between its previously reported antioxidant, antibacterial, and anti-biofilm efficacy, and the related chemical profile, as well as purification of its high features. Afterwards, in vitro activities will be investigated to identify the most promising metabolite(s) and how to optimize their efficacy via nanotechnology.

## 2. Results

### 2.1. Chemical Diversity of Natural Products in OM Extract

**Figure 1:**
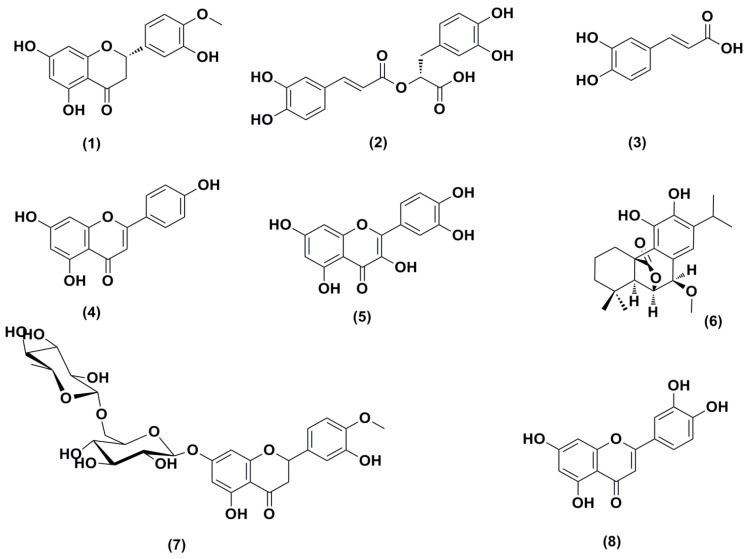
Structures of high features of compounds (ranked by peak intensity) detected in hydromethanolic extract of OM after dereplication of their metabolomes.

The mass resolution in this current study was 50,000 (atm/z 400), which is sufficient to differentiate closely related metabolites. The total number of features found by LC-HRMS in OM extract is documented in Table 1 and Figure S1. The extract with the greatest number of features identified is documented in Table 2 and Figure 1.

### 2.2. Dereplication of OM Extract

In a target-based functional assay, crude hydromethanolic extracts of OM were active as antimicrobials and antioxidants [29,30]. Most of the metabolites from the OM extract were putatively assigned as polyphenolics (Table 1, Figure 1 and Figure S1). Furthermore, several of those were identified as flavonoids, such as hesperitin, apigenin, rutin, and quercetin, which were previously reported in OM [30]. In addition, phenolic acids such as rosmarinic acid, caffeic acid, and ferulic acid [20], and triterpenes such as oleanolic acid and ursolic acid [31], were detected as plausible congeners (Table 1).

### 2.3. Identification of Purified Metabolites

All physical characteristics and ^1^H and ^13^C NMR spectral analysis of purified metabolites are represented in Section S2 of the “Supplementary Material File”.

### 2.4. Biological Evaluation of Purified Compounds

#### 2.4.1. Antimicrobial Activity

**Figure 2:**
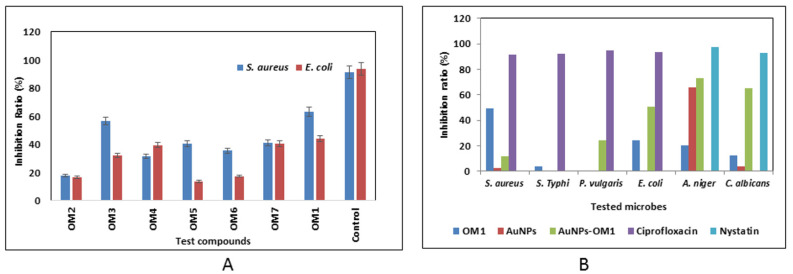
In vitro antimicrobial activity. (**A**) Antibacterial activity of different purified compounds, compared to control (ciprofloxacin). (**B**) Antibacterial and antifungal activity of compound OM_1_, compared with AuNPs and AuNPs-OM_1_.

The *O. majoranum* purified metabolites (high features) were investigated against *E. coli* and *S. aureus* using the MTP assay. Results showed that all purified metabolites displayed low to moderate antimicrobial properties against all tested bacteria, with inhibition ratios ranging from 13.720% to 63.160%. In addition, compound OM_1_ exhibited the highest antibacterial activity with an inhibition ratio of 63.160%. To compare the inhibitory effects of OM_1_ and AuNPs-OM_1_ on the growth of microbes, different bacterial and fungal species were tested. The OM_1_ compound exhibited low to moderate antimicrobial activity toward all tested bacterial and fungal strains, with inhibition ratios ranging from 12.512% and 49.377%. Additionally, the antibacterial activity of OM_1_ was elevated after loading on gold nanoparticles (AuNPs), which caused the increase in the inhibition ratios against *P. vulgaris* and *E. coli* to be 24.419% and 50.658%, respectively. Moreover, AuNPs-OM_1_ exhibited better inhibitory activity against *A. niger* and *C. albicans* fungal strains than OM_1_, individually with inhibition ratios of 73.150% and 65.200%, respectively (Figure 2).

#### 2.4.2. Biofilm Inhibitory Percentage (%) of OM_1_ and AgNPs-OM_1_

Biofilm inhibition activity was examined using microtiter plates. The biofilm inhibition efficiency of the substances OM_1_ and AuNPs-OM_1_ was studied against four clinical pathogenic bacteria (*S. aureus*, *E. coli*, *B. subtilis*, and *P. aeruginosa*), and the biofilms of each of these bacteria were compared to the control (untreated biofilms). In preliminary antibiofilm experiments, the phenolic OM_1_ demonstrated limited antibiofilm activity against all tested bacteria with biofilm inhibitory activity up to 10.552%. Additionally, the AuNPs-OM_1_ reduced the biofilm formation of all strains, especially *E. coli*, by 30.02% (see Table 3).

#### 2.4.3. Antioxidant Activity of the Purified Compounds and AuNPs-OM_1_

The absorbance value at 517 nm shows that compound OM_1_ has the highest DPPH scavenging activity (IC_50_ = 2.41 µg). In contrast, the lowest DPPH depletion was found in the OM_5_, revealing a low antioxidant “power” of this compound. The other phenolics, namely quercetin, rosmarinic acid, caffeic acid, luteolin, and hesperidin, showed similar scavenging activities to OM_1_, ranging from 65.63% to 89.38%. The AuNPs-OM_1_ did not potentiate the antioxidant activity of OM_1_ compared to antimicrobial and antibiofilm activities (scavenging activity = 55.50%) (see Table 4; Table 5).

### 2.5. Gold Nanoparticles’ Preparation and Conjugation with Compound OM_1_

**Figure 3:**
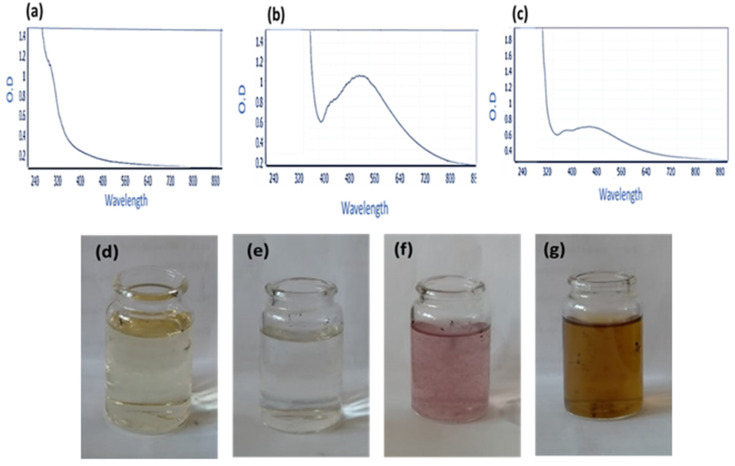
Surface plasmon bands of the compound OM_1_ (**a**), AuNPs (**b**), and the AuNPs-OM_1_ conjugate (**c**). Color change of the gold alone (**d**) and extract (**e**), when mixed together (**f**) and the formation of AuNPs-OM1 (**g**). Surface plasmon absorption bands (SPR).

Based on the obtained antimicrobial, antibiofilm, and antioxidant activity, the compound OM_1_ was selected for loading on gold nanoparticles. Gold nanoparticles (AuNPs) were synthesized utilizing GSH in this study. Creating a covalent bond between the cysteine thiolate of GSH and the gold nanoparticles’ surface in the HAuCl_4_.3H_2_O mediates the synthesis. This interaction caused AuNPs to cluster together on GSH molecules, and the addition of NaBH_4_ at pH 8 resulted in the production of ruby-red AuNPs. The prepared AuNPs using GSH and NaBH_4_ exhibited a characteristic surface plasmon band (SPR) at 520 nm, and the surface plasmon resonance (SPR) absorption spectral range was from 385 to 540 nm. On the other hand, the plasmon band of the conjugate was also measured (Figure 3). These findings were consistent with Sulaiman et al.’s earlier research [32]. The conjugation process between compound OM_1_ and AuNPs was conducted at pH 5. The surface plasmon of the compound OM_1_ alone was measured, and no characteristic surface plasmon bands were measured (Figure 3).

### 2.6. Electron Microscopy

**Figure 4:**
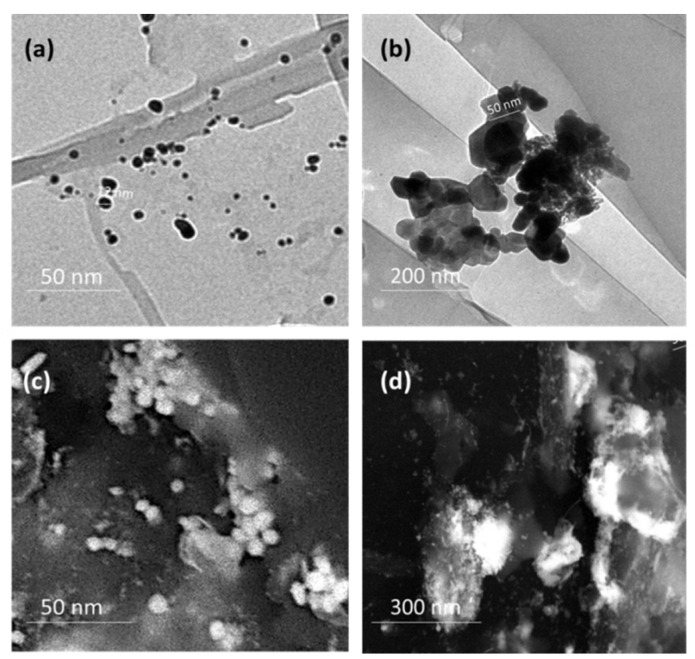
TEM and SEM micrographs of the prepared AuNPs (**a**,**c**) and AuNPs-OM_1_ (**b**,**d**).

The synthesized AuNPs and AuNPs-OM_1_ conjugate morphology and size were investigated using transmission electron microscopy (TEM) and field emission scanning electron microscopy (FESEM). According to the TEM micrograph, the produced AuNPs had an average particle size of approximately 5.02 to 30.20 ± 25 nm, with a spherical shape (Figure 4).

### 2.7. XRD of the Prepared AuNPs

**Figure 5:**
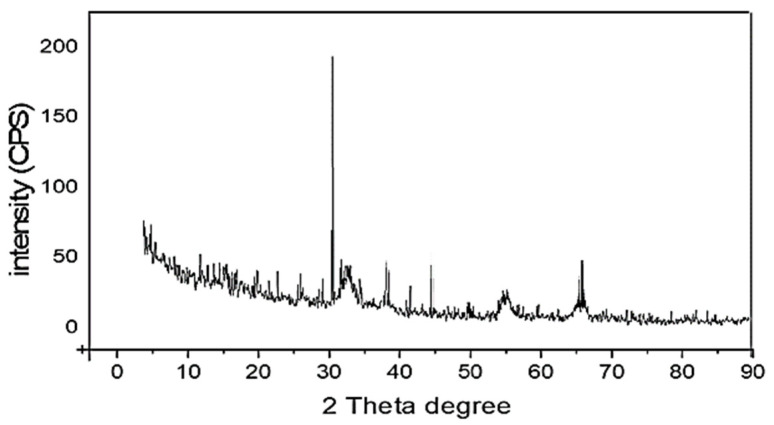
The XRD of the prepared AuNPs.

XRD is considered the most important technique to study the structural properties of the prepared nanomaterials. Therefore, the prepared AuNPs were examined via the XRD diffraction pattern. Figure 5 represents the XRD result of Au nanoparticles. The prepared AuNPs attained in the existence of AuCl_4_^-^ analogous diffraction peaks are assigned to the metallic Au phase with the most essential characteristic peaks, which appeared at 38.0°, 44.2°, and 64.1°, accredited to the crystallographic planes (1 1 1), (2 0 0), and (2 2 0), respectively.

### 2.8. Fourier Transform Infrared Spectroscopy Analysis (FTIR)

**Figure 6:**
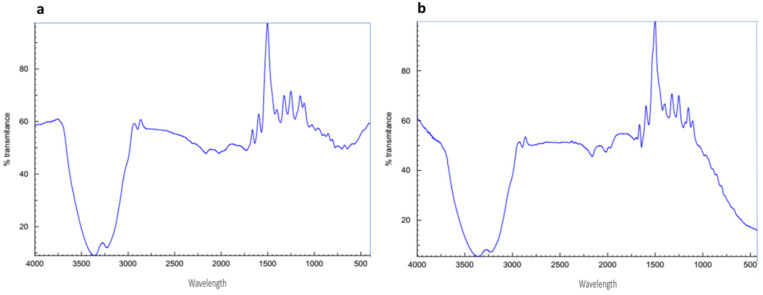
FTIR spectra for compound OM_1_ (**a**) and the prepared AuNPs-OM_1_ (**b**).

For the characterization of functional groups presenting AuNPs and AuNPs-OM_1_, FTIR analysis is required. The FTIR spectra of the compound alone, OM_1_, and AuNPs-OM_1_ were recorded in the spectral region of 4000–400 cm^−1^ and are exhibited in Figure 6.

## 3. Discussion

The topic of oxidative stress and its control by antioxidants is receiving more attention than ever. In nutrition, many consumers and healthcare practitioners closely examine the antioxidant content of typical diet components [33]. The phenolic structure of polyphenols determines their antioxidant activity, and those with catechol-like moieties and the ability to delocalize unpaired electrons have the highest activity. Given the significance of oxidation in several disease pathways and the high antioxidant activity of numerous phenolic compounds in vitro, it was logical to believe that antioxidant activity explained the association between dietary polyphenols and disease prevention [34]. On the other hand, there has been an increase in interest in discovering and producing novel antimicrobial compounds from a variety of sources to address microbial resistance in recent years. Therefore, antimicrobial activity screening and evaluation methodologies have received more attention [35]. Polyphenols found in vegetables and medicinal plants have been studied extensively for their antibacterial action against a variety of pathogens [36].

The in vitro microbicidal activity of the alcoholic extracts of *Origanum majorana* L. was previously tested against diverse fungi such as *Aspergillus niger*, *Fusarium solani*, *Candida albicans*, and *A. parasiticus*, and different bacteria such as *Bacillus subtilis*, *B. megaterium*, *Escherichia coli*, and *Proteus vulgaris*, as well as in vitro antioxidant activity against reactive oxygen species was evaluated. As a result, both antimicrobial and antioxidant assays suggested that the alcoholic extract of *O. majorana* can be used as an effective herbal protectant against different pathogenic bacteria and fungi and has a powerful antioxidant capacity toward various free radicals [29,30,37,38,39,40].

To establish a reason for this result, metabolomics utilizing LC-HRMS and dereplication of *O. majorana* extract were performed to identify various compounds and understand the leading causes of the previously reported antimicrobial and antioxidant potential. According to metabolomics and dereplication, it was clear that *O. majorana* extract possesses a high chemical diversity. In particular, there were eight phenolic compounds identified as high features (high intensity), namely 7-methoxyepirosmanol, rosmarinic acid, quercetin, caffeic acid, hesperitin, luteolin, apigenin, and hesperidin. Most of these metabolites were previously reported in *O. majorana* alcoholic extracts [30,35]. An extensive search of these metabolites concerning their antimicrobial and antioxidant activities revealed that they displayed low to moderate effects against various bacterial and fungal strains [41,42,43], while they demonstrated powerful antioxidant scavenging activity towards ROS [44,45,46], which is highly matched with our results.

Polyphenolic-nanoparticle conjugates have recently been investigated for targeted medication activity augmentation. Nanoparticle-based drug delivery techniques such as vesicular drug delivery (liposomes), nanocrystals, nanoparticles, solid dispersion, and phospholipid complexes have been used to solve the challenges of poor solubility and low bioavailability [47,48,49,50,51].

Gold nanoparticles (AuNPs) have been employed in a wide range of applications due to their highly tunable physicochemical features [52]. Surface plasmon resonance (SPR)—the oscillation of free electrons on the AuNP surface upon infrared radiation [53,54,55]—is definitely the hallmark of all AuNP optical properties. Herein, 7-methoxyepirosmanol displayed the most powerful antimicrobial and antioxidant activities of all those phenolics, and thus it was selected to be loaded on gold nanoparticles to establish activity optimization.

Loading 7-methoxyepirosmanol on nanoparticles exhibited an optimization result for both antimicrobial and antibiofilm activities but not for antioxidant scavenging activity. To our knowledge, gold nanoparticles have not exerted any role in the alteration of the antioxidant capacity of various reducing agents but have been considered analytical tools for antioxidant capacity assessment [56]. Moreover, the antimicrobial and biofilm activity of AuNPs-OM_1_ against several pathogens was substantially (*p* < 0.05) higher than that of free OM_1_. The bactericidal activities of AuNPs-OM_1_ against microorganisms were consistent with the findings of other researchers. In one investigation, azithromycin-loaded nanoparticles outperformed free azithromycin against *S. Typhimurium* [57]. Nisin-loaded nanoparticles inhibited the growth of *Escherichia aerogenes*, *M. luteus*, *P. aeruginosa*, *S. enterica*, and for 20 days, compared to free nisin, which had antibacterial action for 6 days [58]. That effect could be attributed to the smaller particle size, which allows for improved cell penetration and uptake [51].

## 4. Materials and Methods

### 4.1. Plant Material

The leaves of *O. majoranum* (OM) plant were obtained in March 2021 from a field near Elwasta Capital, Beni Suif, Egypt. Professor Abdel-Halim A. Mohammed, Horticultural Research Institute, Department of Flora and Phytotaxonomy Research, Dokki, Cairo, Egypt, certified the plant’s authenticity. A voucher specimen (2021-BuPD 57) was deposited at the Department of Pharmacognosy, Faculty of Pharmacy, Beni Suif University.

### 4.2. Plant Extraction

*O. majoranum* powdered plant (1 kg) was macerated with 80% MeOH at room temperature and then concentrated under reduced pressure using a rotary evaporator (IKA, Königswinter, Germany) to a syrupy consistency. The concentrated methanolic extract yielded 60 g, and the dried extract was stored at 4 °C for in vitro and metabolomic studies.

### 4.3. Metabolomics Analysis

According to Hifnawy et al. [59], the extracted *O. majoranum* powder was subjected to metabolomic analysis using the LC-HRESIMS technique, detailed in Section S1 of the “Supplementary Material File”, representing the HR-MS chart of the main identified components (Figure S1).

### 4.4. Purification of High Features from O. majoranum

#### 4.4.1. Fractionation of the Hydromethanolic Extract

The concentrated methanolic extract of OM (170 gm) was suspended in distilled water (500 mL) and extracted with petroleum ether, DCM, EtOAc, and BuOH, in that order. Under reduced pressure, the organic phase of each step was evaporated individually to afford the corresponding fractions OM-I (0.6 g), OM-II, (35 g) OM-III (18 g), and OM-IV (60 g), respectively. The resulting EtOAc (OM-III) and BuOH (OM-IV) fractions were kept at 4 °C for the phytochemical investigation.

#### 4.4.2. Purification of 7-Methoxyepirosmanol

On a silica gel column (1 × 100 cm, 50 g), a portion of fraction OM-I (600 mg) was fractionated. First, elution was carried out utilizing a petroleum ether-EtOAc gradient mixture in order of increasing polarity (5% to 40%, 10% to 60%, and 20% to 100%), then EtOAc-MeOH (80:20), (50–50), and finally MeOH 100%. The effluents were then collected in test tubes (20 mL), concerning each fraction of 200 mL. Afterwards, each resulted fraction was concentrated and visualized by TLC. Similar fractions were grouped and concentrated under reduced pressure to provide 10 sub-fractions (OMI_1_–OMI_10_). Sub-fraction OMI_5_ was washed several times with chloroform to afford compound OM_1_ (96 mg).

#### 4.4.3. Purification of Flavonoids and Phenolic Acids

On a silica gel column (2.7 × 110 cm, 150 g), a portion of fraction OM-III (15 g) was fractionated. First, elution was carried out utilizing CHCl_3_-EtOAc gradient mixtures in order of increasing polarity (5% to 40%, 10% to 60%, and 20% to 100%), then with EtOAc-MeOH (80:20), (50–50), and finally with MeOH 100%. The effluents were separated into fractions of 400 mL each, which were then divided into test tubes (20 mL), and each fraction was concentrated and monitored using TLC. Six sub-fractions (OMIII1–OMIII6) were created by grouping similar fractions together and concentrating them under reduced pressure. Next, a portion of subfraction OMIII4 (2 g) was fractionated under the same conditions as fraction OM-III, yielding OMIII4-F4 and OMIII4-F5, the latter of which is pure compound OM2 (105 mg). OMIII4-F4 was then subjected to a Sephadex LH-20 column (80 × 1.5 cm, 15 g) using MeOH-H_2_O (8:2) to afford compounds OM_3_ (32 mg), OM_4_ (43 mg), OM_5_ (54 mg), and OM_6_ (102 mg).

On a polyamide-6 column (3.5 × 100 cm, 100 g), a portion of fraction OM-IV (10 g) was fractionated. Afterwards, elution was carried out utilizing MeOH-H_2_O gradient mixtures in order of decreasing polarity (5% to 40%, 10% to 60%, and 20% to 100%). The effluents were collected in various fractions (600 mL each), and each resulted fraction was concentrated and visualized by TLC. To create 16 sub-fractions (OMIV_1_–OMIV_16_), similar fractions were clustered together and concentrated at reduced pressure. OMIV_6_ (200 mg) was then subjected to chromatographic separation using a Sephadex LH-20 column (110 × 1 cm, 15 g) using MeOH-H_2_O (8:2) to yield OMIV_6_-F3, which was afforded to be compound OM_7_ (24 mg). All chemicals, reagents, and apparatus are mentioned in Section S2 of the “Supplementary Material File”.

### 4.5. The Antimicrobial Activity Determination of Phenolic Compounds

To test pure compounds for antibacterial activity, three Gram-negative bacteria (*Proteus vulgaris, Salmonella typhimurium*, and *Escherichia coli* ATCC 25955), one Gram-positive bacteria (*Staphylococcus aureus NRRL B-767*), and two yeasts (*Aspergillus niger* ATCC 16404 and *Candida albicans* ATCC 10231) were used as test organisms and antibacterial tests were performed [60]. The experiments were carried out in 96-well flat polystyrene plates. First, 10 µL of test extracts (final concentration of 250 g/mL) were added to 80 L of lysogeny broth (LB broth), then 10 µL of bacterial culture suspension (log phase) was added, and the plates were incubated overnight at 37 °C. Following incubation, the positive antibacterial action of the tested drug was observed as clearance in the wells. In contrast, compounds that had no effect on the bacteria caused the growth media to become opaque in the wells. Finally, the absorbance was measured after roughly 20 h at OD_600_ in a Spectrostar Nano Microplate Reader (BMG LABTECH GmbH, Allmendgrun, Germany).

### 4.6. Antibiofilm Activity

The 96-well flat polystyrene plates were used to test the biofilm inhibitory activity of compound OM1 and AuNPs-OM1 against four clinical microorganisms, including Gram-positive bacteria (*Staphylococcus aureus* and *Bacillus subtilis*) and Gram-negative bacteria (*Pseudomonas areuginosa* and *Escherichia coli*) [61]. In brief, each well was filled with 180 µL of lysogeny broth (LB broth) and then inoculated with 10 µL of pathogenic bacteria, followed by the addition of 10 µL (final concentration of 250 µg/mL) of samples along with a control (without test sample). The plates were incubated for 24 h at 37 °C, following which the contents in the wells were removed and washed with 200 µL of phosphate buffer saline (PBS), pH 7.2, to remove free-floating bacteria, and then dried in sterilized laminar flow for 1 h. For staining, 200 µL of crystal violet (0.1% *w*/*v*) was applied to each well for 1 h, then the surplus stain was removed, and the plates were retained for drying. Furthermore, dried plates were washed with 95% ethanol, and then optical density was evaluated at an optical density of 570 nm using a Spectrostar Nano Microplate Reader (BMG LABTECH GmbH, Allmendgrun, Germany).

### 4.7. DDPH Antioxidant Assay

The DPPH free radical scavenging experiment was used to assess the antioxidant activity of various metabolites [62]. A fresh DPPH solution in methanol was produced, and the accurate initial concentration was determined spectrophotometrically from a calibration curve (Equation (1)):

ABS_515nm_ = 10,500 × [DPPH] − 1.4 × 10^−2^ (1)

The linear regression (r^2^ = 0.999) suggested that the model was well-fitting. The kinetic measurements for each antioxidant investigated were performed using the spectrophotometer model Cary Bio 100 (Varian, Australia). Moreover, the sample chamber’s temperature was kept under control using a Peltier device incorporated into the chamber. In the literature, DPPH radical scavenging by H atom-donating antioxidants has been described utilizing at least two methods: (a) the fixed reaction time approach and (b) the steady-state saturation method. We tested both strategies to compare their outcomes.

### 4.8. Preparation of Gold Nanoparticles

Gold nanoparticles were prepared as described by Wu et al., [13].50 mL of 0.019 M reduced L-glutathione (GSH) aqueous solution was added to 5 mL of tetrachloroauric acid aqueous solution (0.025 M) and rapidly agitated for 30 min, then NaOH (0.1 M) was used to adjust the pH of the mixture to 8. To get rid of the excess GSH and other salts, the AuNPs were centrifuged for 3 h at 5000 rpm with a freshly prepared aqueous NaBH_4_ (2 mg/mL) under strong stirring until the ruby-red color formed. The supernatant was declined after centrifugation, and the gold nanoparticles were distributed in water before centrifugation was performed again to obtain clean AuNPs.

### 4.9. Characterization of Prepared Nanoparticles

The formation of AuNPs was initially monitored by a color change of the solution. Then, the transition of Au^3+^ to Au^0^ was tracked by regularly sampling aliquots (1 mL) of the mixture and analyzing the UV-vis spectra of the solutions with a SPECTROstar Nano Absorbance Plate Reader (BMG LABTECH). Finally, the gold nanoparticle solution was drop-coated onto a glass substrate, and the X-ray diffraction patterns were recorded using a PANalytical X’pert PRO X-ray diffractometer (The Netherlands) with Cu Ka1 radiation at 40 kV and 30 mA, respectively. Further, the diffracted patterns were captured at 2θ with the scanning speed of 0.02°/min from 10° to 80°. According to Brock-Neely, the ATR-FTIR spectra (Thermo Nicolet 380) of gold nanoparticles were obtained utilizing Broker vertex 80 v in the range of 4000–400 cm^−1^ with a resolution of 4 cm^−1^ (1957) [13].

Transmission electron microscopy (TEM) and scanning electron microscopy were used for analysis of the size and morphology of the produced gold nanoparticles and conjugate. First, 2–4 µL of gold nanoparticle solution was placed on carbon-coated copper grids for sample preparation. Next, the thin film was formed and air-dried under ambient circumstances and detected using Philips 10 Technai with an accelerating voltage of around 180 keV with a wavelength (λ) of 0.0251 Å. Next, scanning electron microscopy (SEM) was used to detect the elemental analysis, with a Field Emission Scanning Electron Microscope (FE-SEM) (Quanta FEG-250, The Netherlands) acceleration voltage of 20 kV, attached with EDAX (energy-dispersive X-ray analysis).

### 4.10. Conjugation of Compound OM_1_ and Gold Nanoparticles

Conjugation of compound OM_1_ and the prepared AuNPs was carried out according to Sulaiman et al. [32], whereby 5 mL of prepared AuNPs was combined with compound OM_1_ (500 μg mL^−1^) and stirred at room temperature overnight. The conjugated AuNP-OM_1_ was centrifuged for 1 h at 10,000 rpm after preparation to eliminate excess OM_1_.

## 5. Conclusions

The present work revealed the antimicrobial and antioxidant effects of the aerial parts of *O. Majoranum*, particularly of its metabolites purified from ethyl acetate and butanol fractions. Furthermore, metabolomic and phytochemical investigations of the plant revealed its ability to accumulate and biosynthesize several secondary metabolites, and primarily phenolics, implying their involvement in *O. majoranum’s* previously reported antibacterial and antioxidant activities. As a result, *O. majoranum’s* previously noted antibacterial ability may be partly attributed to the combined effects of these phytochemicals and/or their synergistic interactions. The antibacterial study confirms that the AuNPs-OM_1_ is more effective at controlling the development of the microorganisms tested and in bacterial biofilm inhibition compared with free OM_1_ (the most active compound). After loading the 7-methoxyepirosmanol in gold nanoparticles, the higher antibacterial activity could be attributed to increased cell penetration and uptake. These discoveries may assist in broadening the potential of this plant in future phytotherapy. Given its dietary supplementation and reported edibility, *O. majoranum* may be considered to protect against a variety of disorders. In the near future, more research into the cellular mechanisms and molecular aspects of *O. majoranum’s* antibacterial and antioxidant properties, as well as its phenolic metabolites, is required.

## Figures and Tables

**Table 1:** LC-HRESIMS analysis of OM extract.

Experimentally Accurate *m*/*z*	Theoretically Accurate *m*/*z*	Quasi-Form	Suggested Formula ^a^	Tentative Identification ^b^
302.0791	302.0790	[M+H]^+^	C_16_H_14_O_6_	Hesperitin
347.0762	347.0760	[M+H]^+^	C_17_H_14_O_8_	Rosmarinic acid
272.0893	272.0896	[M+H]^+^	C_12_H_16_O_7_	Arbutin
457.3670	457.3673	[M+H]^+^	C_30_H_48_O_3_	Oleanolic acid
456.3605	456.3603	[M+H]^+^	C_30_H_48_O_3_	Ursolic acid
170.0217	170.0215	[M+H]^+^	C_7_H_6_O_5_	Gallic acid
181.0495	181.0497	[M+H]^+^	C_9_H_8_O_4_	Caffeic acid
164.0471	164.0473	[M+H]^+^	C_9_H_8_O_3_	*P*-Coumaric acid
194.0578	194.0579	[M+H]^+^	C_10_H_10_O_4_	Ferulic acid
270.0529	270.0528	[M+H]^+^	C_15_H_10_O_5_	Apigenin
164.0812	164.0815	[M+H]^+^	C_10_H_12_O_2_	Trans-2-Hydrocinnamic acid
392.1108	392.1107	[M+H]^+^	C_19_H_20_O_9_	6-*O*-4-Hydroxybenzoylarbutin
290.0792	290.0790	[M+H]^+^	C_15_H_14_O_6_	Catechin
611.1606	611.1609	[M+H]^+^	C_27_H_30_O_16_	Rutin
302.0427	302.0426	[M+H]^+^	C_15_H_10_O_7_	Quercetin
539.0974	539.0975	[M+H]^+^	C_30_H_18_O_10_	Amentoflavone
449.1079	449.1077	[M+H]^+^	C_21_H_20_O_11_	Luteolin 7-*O*-β-D-glucoside
360.1905	360.1907	[M+H]^+^	C_21_H_28_O_5_	7-Methoxyepirosmanol
611.1973	611.1972	[M+H]^+^	C_28_H_34_O_15_	Hesperidin
286.0476	286.0477	[M+H]^+^	C_15_H_10_O_6_	Luteolin

^a^ High-resolution electrospray ionization mass spectrometry (HRESIMS) using XCalibur 3.0 and allowing for M+H/M+Na adduct. ^b^ The suggested compound according to the Dictionary of Natural Products (DNP 23.1, 2021 on DVD) and Reaxys online database.

**Table 2:** High features of compounds (ranked by peak intensity) detected in hydromethanolic extracts of OM after dereplication of their metabolomes.

No.	Accurate *m*/*z*	Suggested Formula ^a^	Quasi-Form	Tentative Detection ^b^	Intensity
1	302.0790	[M+H]+	C_16_H_14_O_6_	Hesperitin	2.2 × 10^4^
2	347.0760	[M+H]+	C_17_H_14_O_8_	Rosmarinic acid	1.2 × 10^7^
3	181.0497	[M+H]+	C_9_H_8_O_4_	Caffeic acid	4.4 × 10^7^
4	270.0528	[M+H]+	C_15_H_10_O_5_	Apigenin	2.3 × 10^7^
5	302.0426	[M+H]+	C_15_H_10_O_7_	Quercetin	8.8 × 10^5^
6	360.1907	[M+H]+	C_21_H_28_O_5_	7-Methoxyepirosmanol	3.6 × 10^6^
7	611.1972	[M+H]+	C_28_H_34_O_15_	Hesperidin	1.1 × 10^4^
8	286.0477	[M+H]+	C_15_H_10_O_6_	Luteolin	6.8 × 10^6^

^a^ High-resolution electrospray ionization mass spectrometry (HRESIMS) using XCalibur 3.0 and allowing for M+H/M+Na adduct. ^b^ The suggested compound according to the Dictionary of Natural Products (DNP 23.1, 2021 on DVD) and Reaxys online database.

**Table 3:** Biofilm inhibitory percentage (%) of OM_1_ and AgNPs-OM_1_.

Biofilm Inhibitory Percentage (%)				
Test Bacteria	*S. aureus*	*B. subtilis*	*P. aeruginosa*	*E. coli*
Compound OM_1_	5.245	7.025	0	10.552
AuNPs-OM_1_	19.251	15.551	0	30.021

**Table 4:** Scavenging activity (%) of the purified compounds and AuNPs-OM_1_.

Sample (100 µL) (Concentration = 4 µg)	Scavenging Activity (%)
OM_1_	91.59
OM_2_	89.38
OM_3_	76.078
OM_4_	68.88
OM_5_	58.58
OM_6_	68.83
OM_7_	65.63
AuNPs-OM_1_	55.50
Ascorbic acid	99.86

**Table 5:** Scavenging activity (%) of the compound OM_1_ at different concentrations.

OM_1_	O.D_517nm_	Scavenging Activity (%)
Control	2.759	
50 µL (1 µg)	2.4145	12.48
100 µL (2 µg)	1.83	33.67
200 µL (4 µg)	0.7114	74.22
300 µL (6 µg)	0.2936	89.35
IC_50_ = 2.41 µg		

## Data Availability

Not applicable.

## Supplementary Materials

The following supporting information can be downloaded at: https://www.mdpi.com/article/10.3390/plants11141871/s1, Figure S1: HRESI/MS spectrum for *O. majoranum* in the positive ionization mode. Figure S2: ^1^H NMR spectrum of compound OM1 (400 MHZ, methanol-d4). Figure S3: ^13^C NMR spectrum of compound OM1 (100 MHZ, methanol-d4). Figure S4: ^1^H NMR spectrum of compound OM_2_ (400 MHZ, methanol-d4). Figure S5: ^1^H NMR spectrum of compound OM_3_ (400 MHZ, DMSO-*d6*). Figure S6: ^1^H NMR spectrum of compound OM_4_ (400 MHZ, methanol-d4). Figure S7: ^1^H NMR spectrum of compound OM_5_ (400 MHZ, methanol-d4). Figure S8: ^1^H NMR spectrum of compound OM_6_ (400 MHZ, methanol-d4). Figure S9: ^1^H NMR spectrum of compound OM_7_ (400 MHZ, DMSO-*d6*). Section S1: Metabolomics analysis. Section S2: Identification of purified metabolites.
