# Supplementary material for: LC/MS Profiling and Gold Nanoparticle Formulation of Major Metabolites from Origanum majorana as Antibacterial and Antioxidant Potentialities
Source: Plants (Basel). 2022 Jul 18;11(14):1871. doi: 10.3390/plants11141871 (PMC9319600; doi:10.3390/plants11141871)
Supplement: Supplementary file 1 [file plants-11-01871-s001.zip › plants-1746347-supplementary.pdf]

plants

Supplementary material

S1. Metabolomics analysis

The metabolomic study of *O. majoranum* crude hydromethanolic extract was performed using LC-HR-ESI-MS analytical methods [59]. In brief, the whole extract (1 mg/mL in MeOH) was uploaded on an Accela HPLC (Thermo Fisher Scientific, Bremen, Germany) paired with Accela UV–vis and Exactive (Orbitrap) mass spectrometer from Thermo Fisher Scientific (Bremen, Germany). The mobile phase consisted of HPLC grade water (A) and acetonitrile (B), each containing 0.1% formic acid. The gradient elution began at a flow rate of 300 μL/min with 10% B and climbed linearly to 100% B after 30 minutes, remaining isocratic for the next 5 minutes before linearly falling back to 10% B for the final 1 minute. Before the second injection, the mobile phase was equilibrated for 9 minutes. For ESI-MS utilizing an in-source CID (collision-induced dissociation) mechanism, the mass range was set to 100–2000 m/z, and for MS/MS using an untargeted HCD mechanism, the mass range was set to 50–1000 m/z (high energy collision dissociation).

In MZmine, the RAW data is imported by selecting the ProteoWizard-converted positive or negative files in mzML format (Raw data methods → Raw data import). The peaks in the samples and blanks were detected using the chromatogram builder. Mass ion peaks were isolated (Raw Data Methods → Peak detection → Mass detection) with a centroid detector threshold that was greater than the noise level set to 1.0×10^4 and an MS level of 1. Following this, the chromatogram builder (Raw Data Methods → Peak detection → Chromatogram builder) was used with a minimum time span set to 0.2 min, and the minimum height and m/z tolerance to 1.0×10^4 and 0.001 m/z or 5.0 ppm, respectively. For all remaining steps, select all files under peak lists before executing each step.

Chromatogram deconvolution was then performed to detect the individual peaks (Peak List Methods → Peak detection → Chromatogram deconvolution). The local minimum search algorithm (chromatographic threshold: 95%, search minimum in RT range: 0.4 min, minimum relative height: 5%, minimum absolute height: 3.0 × 104, minimum ratio of peak top/edge: 3, and peak duration range: 0.2–5 min) was applied. Isotopes were also identified (Peak list methods → Isotopic peaks grouper → Deisotope) using the isotopic peaks grouper (m/z tolerance: 0.001 m/z or 5.0 ppm, retention time tolerance: 0.1 absolute (min), maximum charge: 2, and representative isotope: most intense). This step will only deisotope peaks that were detected in the original search i.e., those assigned a peak ID.

Filtering is useful to set certain parameters when only considering a certain RT window e.g., 5–40 min or m/z range window or to discard IDs that are only present in one sample (Peak List Methods → Filtering → Peak List Rows Filtering). For chromatographic alignment and gap-filling (Peak List Methods → Alignment → Join aligner), the retention time normalizer (m/z tolerance: 0.001 m/z or 5.0 ppm, retention time tolerance: 0.5 absolute (min), and minimum standard intensity: 5.0×10^3) was used to reduce inter-batch variation. The peak lists were all aligned using the join aligner parameters set to m/z tolerance: 0.001 m/z or 5.0 ppm, weight for m/z: 20, retention time tolerance: 5.0 relative (%), weight for RT: 20. The values for the weight of m/z and RT should be kept the same; this means that both RT and m/z are given equal importance.

Missing peaks (peaks undetected by previous algorithms due to deficient peak detection or a mistake in peak list alignments) were detected using the gap filling peak finder (Peak List Methods → Gap filling: Peak Finder) with an intensity tolerance of 25%, m/z tolerance of 0.001 m/z or 5.0 ppm, and retention time tolerance of 0.5 absolute (min). After this step a file will be created called "neg-gap filled" if negative mode and "pos-gap filled" if positive mode. Open the files and after gap-filling delete all peaks found in solvent blanks above a threshold (determined by user).

An adduct search (Peak list methods → Identification → Adduct search) was performed for Na–H, K–H, NH4, formate, and ACN + H (RT tolerance: 0.2 absolute (min), m/z tolerance: 0.001 m/z or 5.0 ppm, max relative adduct peak height: 30%). Additionally, a complex search (Peak list methods → Identification → Complex search) was performed (ionization method: [M + H]+ for ESI positive mode and [M − H]− for ESI negative mode, retention time tolerance: 0.2 absolute (min), m/z tolerance: 0.001 m/z or 5.0 ppm, and with maximum complex peak height of 50%). The processed data set was then subjected to molecular formula prediction and peak identification (Peak List Methods → Identification → Formula Prediction) to search for unidentified features. Select atoms C,H,N,O and any other elements. Adjust parameters with heuristics element count with all three sub-options to get the isotope pattern filter working with all features with isotope peaks.

Excel macros were written to enable the subtraction of background peaks and to combine positive and negative ionization mode data files generated by MZmine. Peaks originating from the solvents were extracted. By applying an algorithm to calculate the intensity of each m/z in tested extract, ion peaks originating from the medium were subtracted while features with peak intensity 20 times greater in the samples than in the medium were retained. The positive and negative ionization mode data sets from each of the respective tested extract were combined by the macro enabling ion peaks that were observed in either or both positive and negative modes to be overlaid for further statistical analysis. The Excel macro was used to dereplicate each m/z ion peak with compounds in the customized database (using RT and m/z threshold of ±3 ppm) which provided details on the putative identities of all metabolites in tested extract and sequentially sorted the number of remaining unknowns for each extract. The macro was then utilized to identify the top 20 features (ranked by peak intensity) and corresponding putative identities in tested sample by creating a list for it. Hits from the database were accessed using ChemBioFinder, Dictionary of Natural Products (DNP 23.1, 2015 on DVD), and Reaxys online database.

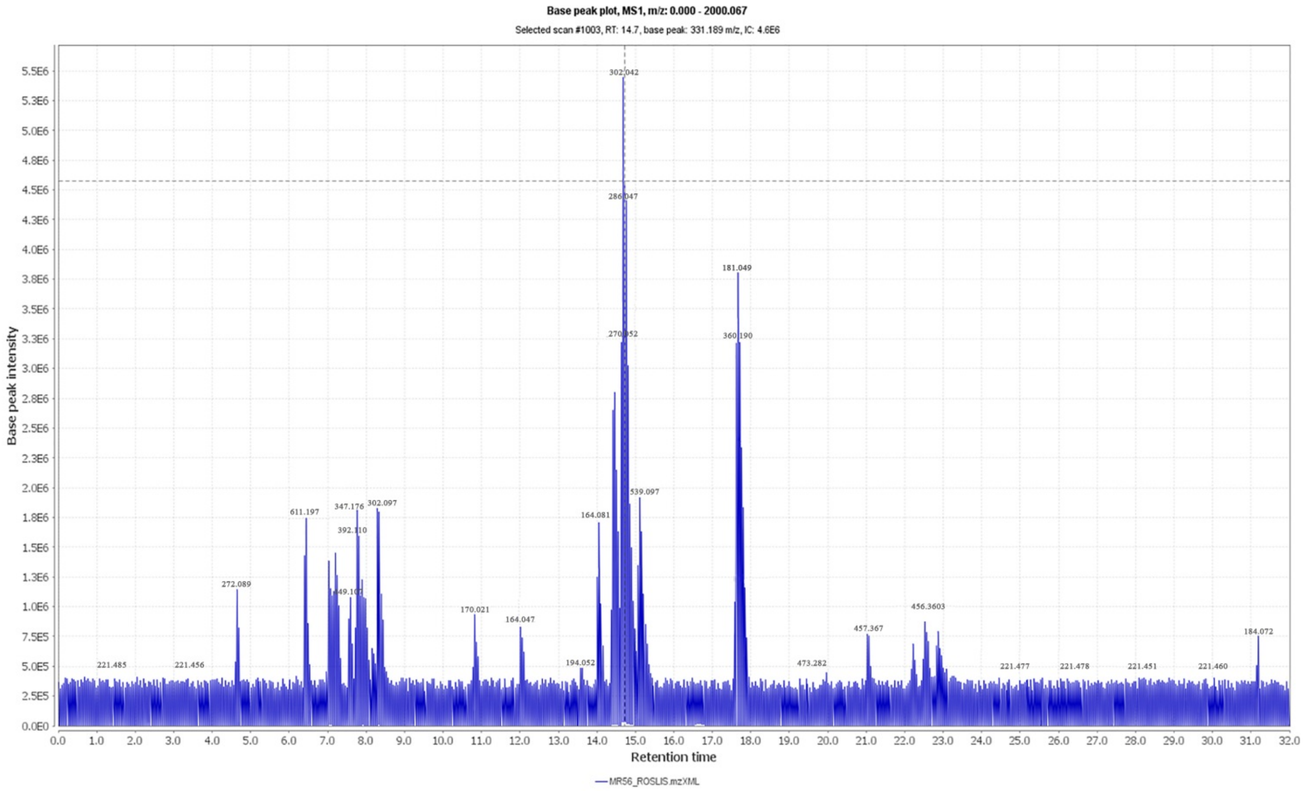

Figure S1. HRESI/MS spectrum for *O. majoranum* in the positive ionization mode.

S2. Identification of purified metabolites

S2.1. Identification of OM_1 (7-Methoxyepirosmanol)

White crystals, M.P: 673.6±55.0 °C. ^{1}H-NMR (400 MHz, methanol-d4) δ_H 1.98 (1H, m, H1-α), 3.32 (1H, m, H1-β), 1.46 (1H, m, H2-α), 1.54 (1H, m, H2-β), 1.28 (1H, m, H2-α), 2.19 (1H, s, H5), 4.29 (1H, d, J = 4 Hz, H6-α), 4.82 (1H, d, J = 4 Hz, H7-α), 6.78 (1H, s, H-14), 3.22 (1H, m, J = Hz, H-15), 3.67 (1H, s, 7-OMe), 1.21 (2H, dd *overlapped*, J = 4, H-16 & H-17), 1.04 (3H, s, H-18) and 0.94 (3H, s, H-19). δ_C 18.78 (C-2), 21.01 (C-19), 21.56 (C-17), 21.76 (C-16), 26.52 (C-15), 27.23 (C-1), 30.66 (C-18), 31.00 (C-4), 37.97 (C-4), 48.47 (C-10), 55.88 (C-5), 57.16 (O-Me), 47.65 (C-7), 77.56 (C-6), 119.46 (C-14), 123.69 (C-9), 126.29 (C-8), 135.96 (C-13), 142.33 (C-12), 143.98 (C-11), 179.54 (C-20). See Figure S2 and Figure S3.

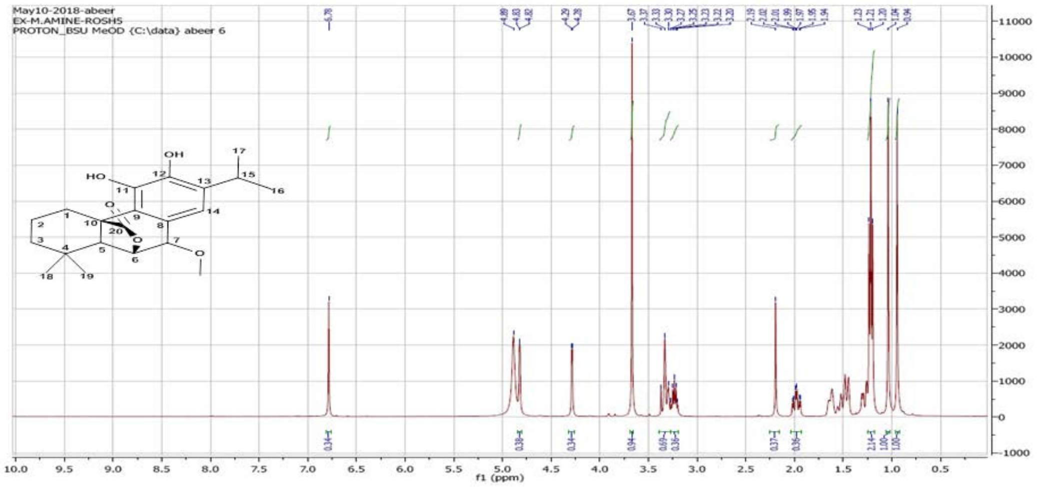

Figure S2. ^{1}H NMR spectrum of compound OM1 (400 MHZ, methanol-d4).

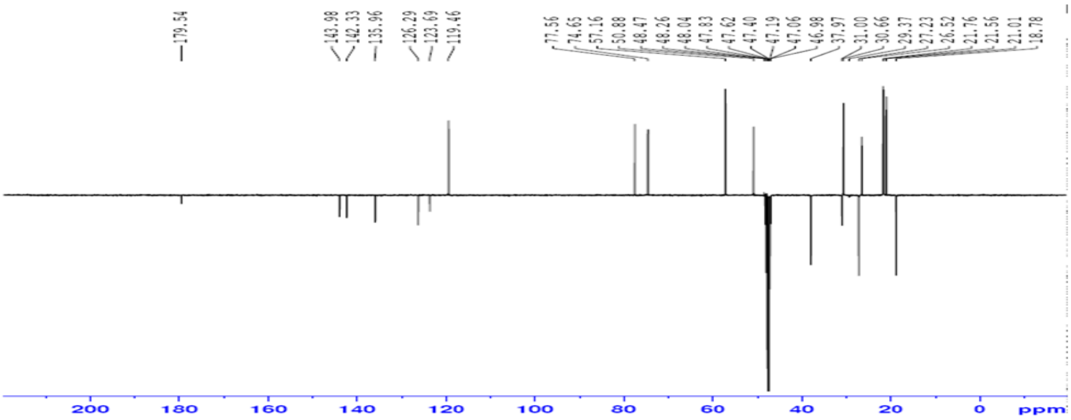

Figure S3. ^{13}C NMR spectrum of compound OM1 (100 MHZ, methanol-d4).

S2.2. Identification of OM_2 (rosmarinic acid)

Orange crystals, M.P: 171-175°C. ^{1}H-NMR (400 MHz, methanol-d4) δH 7.55 (1H, d, J = 15.9 Hz; H-7), 7.04 (1H, br s; H-2), 6.95 (1H, d,J = 7.7 Hz; H-6), 6.78 (1H, d, J = 7.7 Hz; H-5), 6.75 (1H, s; H-2′), 6.70 (1H, d, J = 7.8 Hz; H-5′), 6.62 (1H, d, J = 7.8 Hz; H-6′), 6.26 (1H, d, J = 15.9 Hz; H-8), 5.19 (1H, br d, J = 3.7 Hz; H-8′), 3.10 (1H, br d, J = 13.4 Hz; H-7′a), 3.01 (1H, m; H-7′b). See Figure Figure S4.

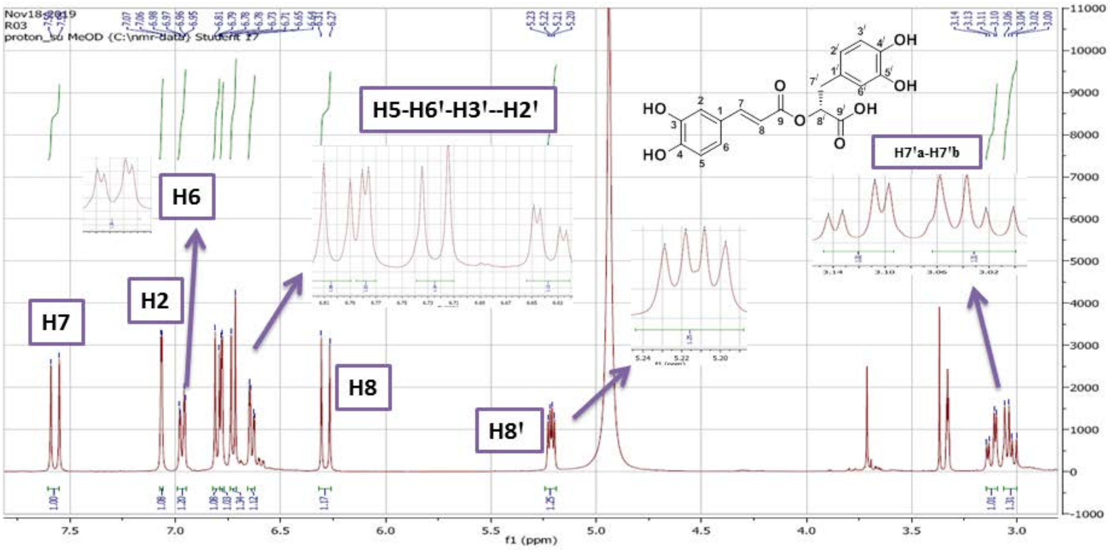

Figure S4. ^{1}H NMR spectrum of compound OM2 (400 MHZ, methanol-d4).

S2.3. Identification of OM$_3$ (Quercetin)

Yellow needles, M.P. 316.5 °C. ^{1}H-NMR (400 MHz, (DMSO-*d6*) δ_H 12.84 (1H, s, OH-3), 10.73 (1H, s, OH-5), 9.53 (1H, s, OH-7), 9.25 (1H, s, OH-4'), 9.29 (1H, s, OH-3'), 7.68 (1H, d, *J* = 2.4, H-6'), 7.55 (1H, dd, *J* = 2.4, 10.4, H-2'), 6.89 (1H, d, *J* = 8.4, H-3'), 6.53 (1H, d, *J* = 2, H-8), 6.27 (1H, d, *J* = 2, H-6). See Figure Figure S5.

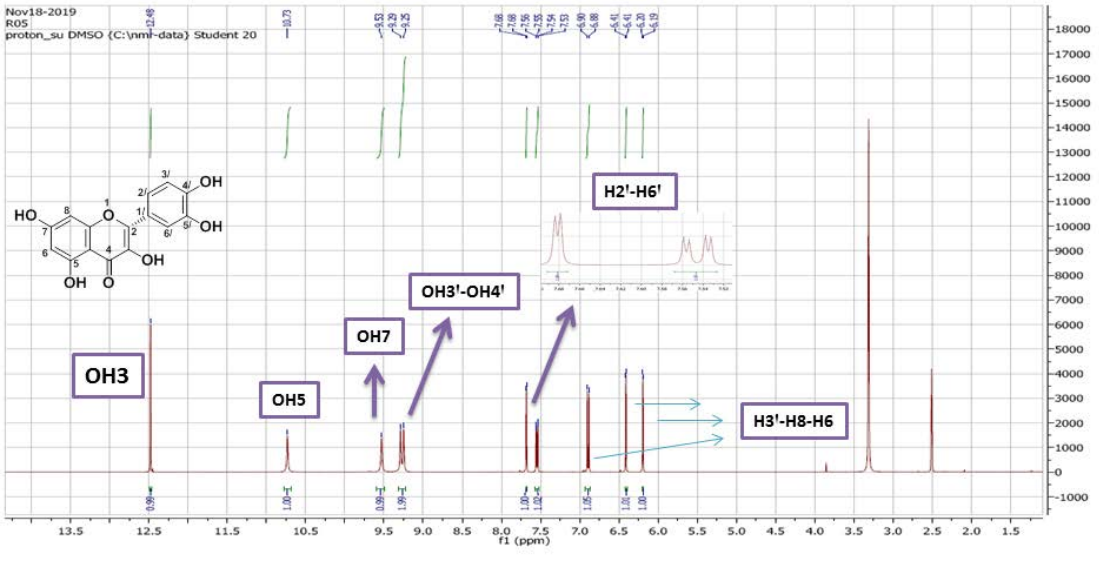

Figure S5. ^{1}H NMR spectrum of compound OM$_3$ (400 MHZ, DMSO-*d6*).

S2.4. Identification of OM$_4$ (caffeic acid)

Yellow amorphous solid, M.P. 225°C. ^{1}H-NMR (400 MHz, methanol-d4) δ_H 7.43 (1 H, d, *J* = 15.6 Hz, H-7), 6.94 (1 H, d, *J* = 2.0 Hz, H-2), 6.84 (1 H, dd, *J* = 8.0, 2.0 Hz, H-6), 6.68 (1 H, d, *J* = 8.4, H-5), 6.21 (1 H, d, *J* = 16 Hz, H-8). See Figure S6.

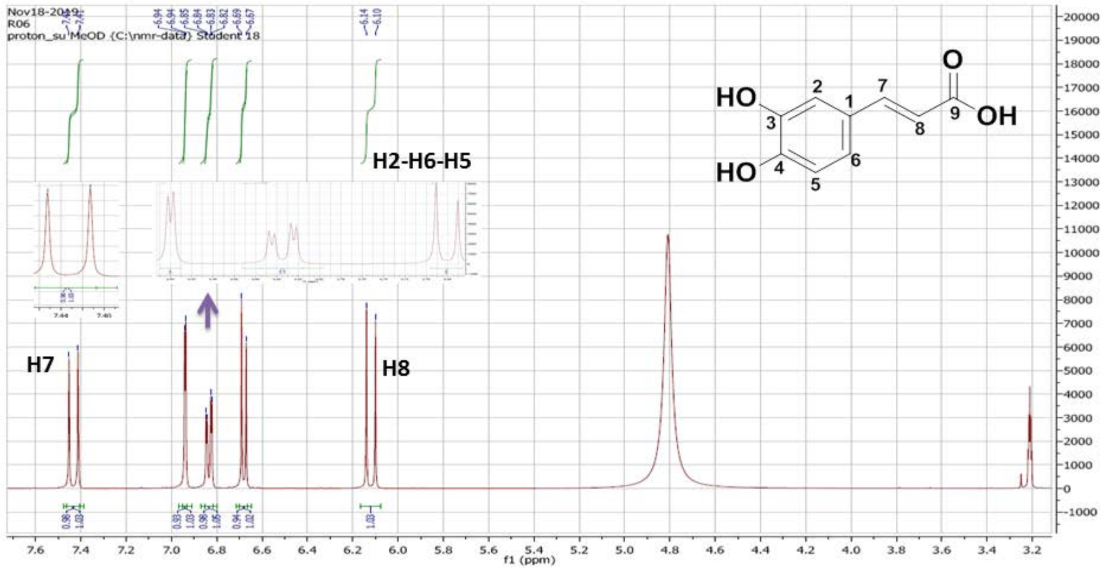

Figure S6. ^{1}H NMR spectrum of compound OM_4 (400 MHZ, methanol-d4).

S2.5. Identification of OM_5 (hesperitin)

Yellow powder, M.P. 227.5 °C. ^{1}H-NMR (400 MHz, methanol-d4) δ_H 2.62 (1H, dd, *J* = 2.8, 16.8 , H-3α), 2.97 (1H, dd, *J* = 12.8, 17.2, H-3β), 3.76 (3H, s, OCH_3), 5.22 (1H, dd, *J* = 12.8, 3.2, H-2), 5.78 (1H, d, *J* = 2.0, H-6), 5.81 (1H, d, *J* = 2.0, H-8), 6.81 (1H, dd, *J* = 8.4, 2.0, H-2′), 6.83-6.85 (2H, *overlapped*, H-6′ & H-3′**)**. See Figure S7**.**

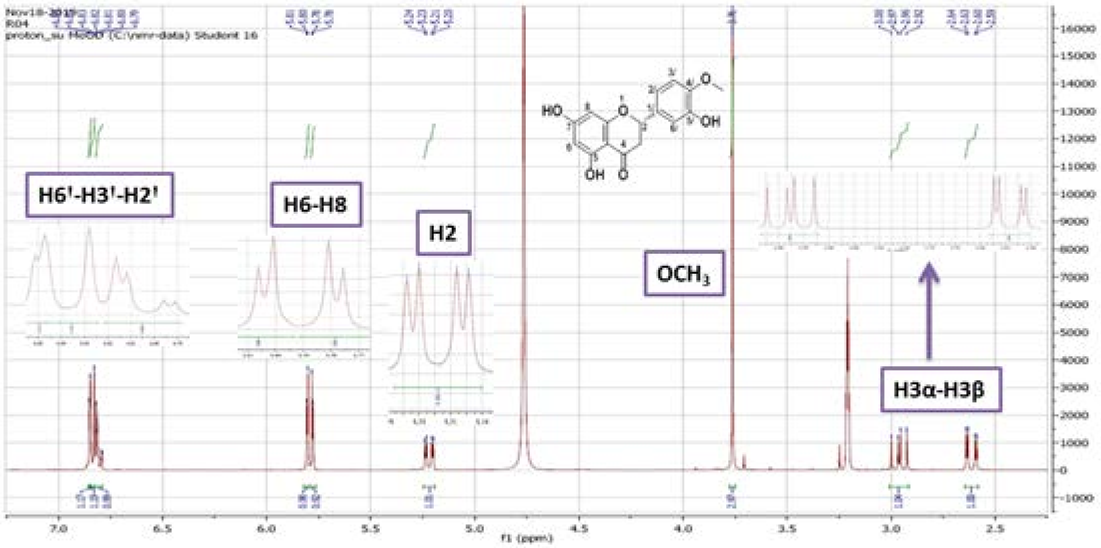

Figure S7. ^{1}H NMR spectrum of compound OM_5 (400 MHZ, methanol-d4).

S2.6. Identification of OM_6 (luteolin)

Yellow powder, M.P. 328-330°C. ^{1}H-NMR (400 MHz, methanol-d4) δ_H 6.23 (lH, *d*, *J* = 2 Hz, H-6), 6.46 (lH, *d*, *J* = 2 Hz, H-8), 6.65 (1H, s, H-3), 6.92 (lH, d, *J* = 8 Hz, H-5′), 7.39-7.41 (2H, *overlapped*, H-2′ & H-6′). See Figure S8.

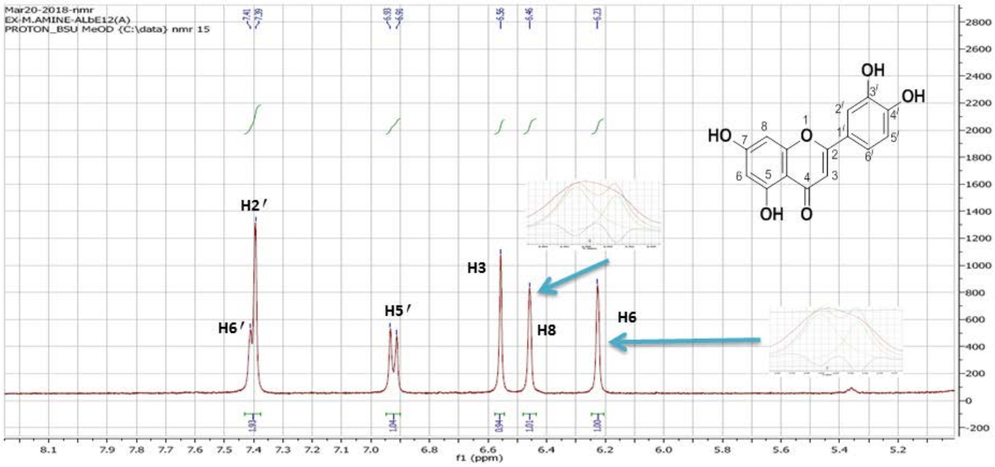

Figure S8. ^{1}H NMR spectrum of compound OM_6 (400 MHZ, methanol-d4).

S2.7. Identification of OM_7 (hesperidin)

Yellow powder, M.P. 262.0°C. ^{1}H-NMR (400 MHz, DMSO-*d6*) δ_H 11.99 (1H, s, OH-5), 9.09 (1H, s, OH-3′), 6.90-6.96 (3H, m, *overlapped*, H-2′, H-5′ and H-6′), 6.14 (1H, d, *J* = 2 Hz, H-6), 6.13 (1H, d, *J* = 2.4 Hz, H-8), 5.50 (1H, dd, *J* = 3.6 and 7.6 Hz, H-2), 4.99 (1H, d, *J* = 8 Hz, H-1 glucose), 4.53 (1H, d, *J* = 6 Hz, H rhamnose), 3.78 (3H, s, CH_3-4′), 3.10–3.28 (m, OH-rhamnoglucosyl and H-3 trans), 2.78 (1H, d, *J* = 3 Hz, H-3 cis), 1.08 (3H, d, *J* = 6.4 Hz, CH_3-rhamnose). See Figure S9.

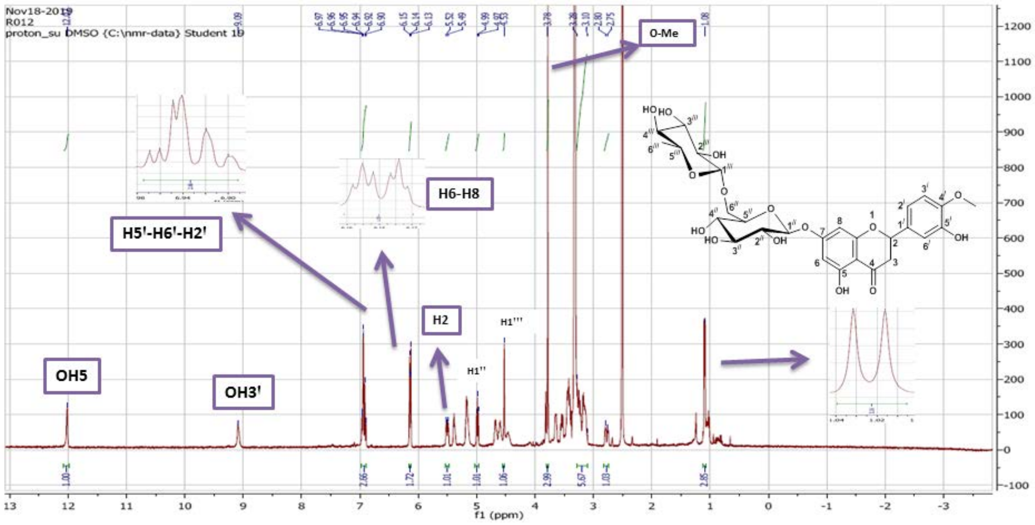

Figure S9. ^{1}H NMR spectrum of compound OM_7 (400 MHZ, DMSO-*d6*).

S2.8. Chemicals and reagents

Solvents used in this work, e.g., petroleum ether (pet. ether; B.p. 60±80ÊC), dichloromethane (DCM), ethyl acetate (EtOAc), methanol (MeOH), and ethanol (EtOH), were purchased from El-Nasr Company for Pharmaceuticals and Chemicals, Egypt, and were distilled before use. Solvents of high performance liquid chromatography (HPLC) grade, e.g., acetonitrile and water were used for HPLC separations and purifications, and were obtained from SDFCL sd Fine-Chem Limited, India. Deuterated solvents (Sigma-Aldrich, Germany), including methanol (CD_3OD) and dimethyl sulfoxide (DMSO-*d6*), were used for nuclear magnetic resonance (NMR) spectroscopic analyses. Column chromatography (CC) was performed using silica gel 60 (E. Merck, Darmstadt, Germany; 60±120 mesh) or sephadex LH±20 (0.25±0.1 mm, GE Healthcare, Sweden). Thin layer chromatography

(TLC) analyses were carried out using pre-coated silica gel 60 GF254 plates (E. Merck, Darmstadt, Germany; 20 × 20 cm, 0.25 mm in thickness). Spots were visualized by spraying with 10% sulfuric acid in methanol followed by heating at 110ÊC. Ammonia vapors and aluminum chloride reagent (5% in ethanol) were also used for detection of flavonoids on TLC, while ferric chloride reagent (1% in ethanol) was used for phenolic compounds. All chemicals used for the preparation of different spraying were obtained from El-Nasr Company for Pharmaceuticals and Chemicals, Egypt.

S2.9. Apparatus

Ultraviolet lamp (UVP, LLC, USA) was used for visualization of spots on thin layer chromatograms at 254 and/or 365 nm. ^{1}H (400 MHz) as well as ^{13}C NMR (100 MHz) and distortionless enhancement by polarization transfer (DEPT-Q; 100 MHz) spectra were recorded on Bruker Avance 400 MHz instruments in DMSO-d6 and CD_3OD. Chemical shift values (δ) were recorded in ppm units and coupling constants (J) in Hz. Solvent signals of DMSO-d6 ($^{\delta}H$ 2.5 ppm and $^{\delta}C$ 39.5 ppm) and CD_3OD ($^{\delta}H$ 3.3 ppm and $^{\delta}C$ 49.0 ppm) were considered as the internal reference signals for calibration. Electrospray ionization mass spectrometry (ESI±MS) spectra were obtained using a Synapt G2 HDMS QTOF (quadrupole time-of-flight)-mass spectrometer (Waters, Germany). HPLC analysis was performed on an analytical Gemini-NX RP-18 column (5 μm, 4.60 × 100 mm; Phenomenex, Germany).
